# Defining ecological regions in Italy based on a multivariate clustering approach: A first step towards a targeted vector borne disease surveillance

**DOI:** 10.1371/journal.pone.0219072

**Published:** 2019-07-03

**Authors:** Carla Ippoliti, Luca Candeloro, Marius Gilbert, Maria Goffredo, Giuseppe Mancini, Gabriele Curci, Serena Falasca, Susanna Tora, Alessio Di Lorenzo, Michela Quaglia, Annamaria Conte

**Affiliations:** 1 Istituto Zooprofilattico Sperimentale dell’Abruzzo e del Molise G. Caporale, Campo Boario, Teramo, Italy; 2 Spatial Epidemiology Lab (SpELL), Université Libre de Bruxelles, Brussels, Belgium; 3 Department of Physical and Chemical Sciences, University of L’Aquila, L’Aquila, Italy; 4 Center of Excellence in Telesensing of Environment and Model Prediction of Severe Events (CETEMPS), University of L’Aquila, L’Aquila, Italy; Faculty of Science, Ain Shams University (ASU), EGYPT

## Abstract

Ecoregionalization is the process by which a territory is classified in similar areas according to specific environmental and climatic factors. The climate and the environment strongly influence the presence and distribution of vectors responsible for significant human and animal diseases worldwide. In this paper, we developed a map of the eco-climatic regions of Italy adopting a data-driven spatial clustering approach using recent and detailed spatial data on climatic and environmental factors. We selected seven variables, relevant for a broad set of human and animal vector-borne diseases (VBDs): standard deviation of altitude, mean daytime land surface temperature, mean amplitude and peak timing of the annual cycle of land surface temperature, mean and amplitude of the annual cycle of greenness value, and daily mean amount of rainfall. Principal Component Analysis followed by multivariate geographic clustering using the k-medoids technique were used to group the pixels with similar characteristics into different ecoregions, and at different spatial resolutions (250 m, 1 km and 2 km). We showed that the spatial structure of ecoregions is generally maintained at different spatial resolutions and we compared the resulting ecoregion maps with two datasets related to Bluetongue vectors and West Nile Disease (WND) outbreaks in Italy. The known characteristics of *Culicoides imicola* habitat were well captured by 2/22 specific ecoregions (at 250 m resolution). *Culicoides obsoletus/scoticus* occupy all sampled ecoregions, according to its known widespread distribution across the peninsula. WND outbreak locations strongly cluster in 4/22 ecoregions, dominated by human influenced landscape, with intense cultivations and complex irrigation network. This approach could be a supportive tool in case of VBDs, defining pixel-based areas that are conducive environment for VBD spread, indicating where surveillance and prevention measures could be prioritized in Italy. Also, ecoregions suitable to specific VBDs vectors could inform entomological surveillance strategies.

## Introduction

Ecoregions have been defined as areas “*within which there are associations of interacting biotic and abiotic features*” [1]. Climate, orography, geological factors (abiotic) and vegetation (biotic), are the characteristics commonly used to define homogeneous land units, within which natural communities and species interact with the physical elements of the environment [2]. Ecoregions are typically applied in forest conservation [3], land management programs [4] and in environmental conservation strategies [2].

Ecoregionalization is then the process through which a territory is classified into similar areas according to specific environmental and climatic factors. There are several datasets already existing for Italy that reflect vegetation distribution (e.g. the CORINE Land Cover (CLC) map [5]), and climatic conditions or bioclimatic indices [6,7], but these datasets are still not fully integrated together. For Italy, the most recent classification available derives from a hierarchical, deductive and divisive expert-based method, vegetation-oriented, that, at its finer scale, divides the Italian land in 33 areas, homogeneous by climate, physiography, biogeography and vegetation [2]. This classification has been further evolved into an ecosystem mapping of Italy, where it was integrated with the CLC and the potential natural vegetation through an expert-based overlay [8].

A quantitative and objective classification (nor hierarchical or supervised) that includes land use, climatic and topographic features, which allows the identification of homogeneous areas at finer scale, using a raster-based approach is still missing. The recent and rapid advances in the performance and accessibility of computer hardware and statistical software facilitate the ecoregionalization process, taking also advantage of the large volume of spatial data availability, also remotely sensed [9].

Whether remotely sensed or ground collected, ancillary or in real time, the selection of data sets to be included in an ecoregionalization process responds to the specific purpose for which the similarity analysis is conducted.

In addition to the traditional fields of application explored so far, the use of the ecoregionalization approach can be of interest also in the veterinary field especially when dealing with vector-borne diseases, where the triangle vectors-host-pathogen is strictly linked to the environment [10–13]. Suitable conditions allow the proliferation of the vectors, providing rest and breeding sites, shapes the pathogen replication and facilitates the contact among the three actors promoting the infectious disease transmission. A recent assessment indicated that there is a common set of drivers to many human and domestic animal pathogens; in particular, the 82% of the most significant human and domestic animal pathogens occurring in Europe are influenced by temperature, rainfall, humidity or wind, and this is particularly true for VBDs [14].

Given the presence of susceptible reservoir hosts and competent vectors, areas with similar climatic and environmental conditions, are potentially exposed to similar disease risk, even if they are geographically very distant from each other. This simple assumption underlines much of the spatial modelling work that has been developed so far. Numerous studies have earlier examined the complex interactions among environment and vectors/diseases [15–18], also through the species distribution models (SDM) applied to vector occurrence [18–22]. They are then useful when the vectors are known and their distribution enough assessed with reliable field dataset so to feed the model and produce accurate predictions. When an incursion of a new VBD is observed in an area, species not previously known to be competent vector could result implicated in the transmission of the disease, as happened with bluetongue BTV8 in Northern Europe in 2006 [23,24] and this can limit the application of SDM so far developed. Emerging vector borne diseases could therefore benefit from a different approach starting from the definition of generic ecoregions, mapping the climatic and environmental similarity so to inform the spatial context in which a VBD can potentially spread or persist. The idea of identifying similar areas in terms of climatic and environmental conditions does not aim to link a specific vector to specific conditions, but rather to highlight where the same characteristics (e.g. high/low temperatures, heavy/light rain, dense/sparse vegetation, etc.) are present so as to be able to identify *a priori* where a possible vector that prefers those specific conditions could spread.

In this paper, we identified similar eco-climatic regions of Italy following a data-driven spatial clustering approach and using the available, most detailed and up-to-date spatial data on climatic and environmental factors. The variables chosen, although not exhaustive, are among those that have been repeatedly found to be associated with a set of human and animal diseases [14], in particular with Bluetongue [25,26] and West Nile [27–29], two vector borne diseases affecting Italy in the last decades.

We also compare the resulting ecoregion maps with two dataset, i) the distributions of some BT vectors and ii) the distribution of WND outbreaks, to highlight pros and cons of such an approach.

## Materials and methods

### Environmental and climatic selected data

Seven variables related to topography, temperature, rainfall and vegetation, and known to be relevant to VBDs, available for the entire Italian territory, were selected for the ecoregionalization process.

Topography influences the water runoff and the retention of water and nutrients, affecting the moisture of soil surface layer. Depending on the vector preferences for a mud, moist or aquatic breeding site, the topography contributes to promote or inhibit the proliferation of larval sites [17,30,31].

Temperature influences the vector population dynamics and it drives the vector competence, by accelerating the virus replication within the insects and prolonging their breeding season [32,33].

The effect of rainfall is more controversial in literature and is highly dependent on the habits of the vector species. Water pools are fundamental for the larval stage of mosquitoes and rainfall events favour their proliferation, but at the meantime dilute the content of nutrients decreasing their reproduction rate [16,34]. Other vector species, as *Culicoides imicola*, needs moist soil but not flooded to breed [17], and high rainfall events could eliminate larval habitats and create unsuitable environmental conditions.

Vegetation is a key parameter both to define vector habitat, and as a proxy of rainfall and humidity. The Normalised Difference Vegetation Index (NDVI) is a wide used index in remote sensing [35], representing the presence and density of green biomass. It is widely present in epidemiological models to explain disease occurrences [36–38] or to describe the vector habitats [25,39–41].

In our study, temperature and vegetation were derived from remotely sensed archives and submitted to a Fourier decomposition: yearly time series in each pixel is decomposed in sine curves (harmonics), each characterised by specific amplitudes and phases whose sum recompose the original dataset [42]. Usually the first two harmonics are retained, as they explain the majority of variability and are easily biologically interpretable. The first component of the series, the harmonic 0, is an average of the values across each year and it gives the yearly mean value of the variable (A0). The harmonic 1 is characterised by amplitude (A1) and phase (ph1) and it describes the annual cycle of the variable. Once the yearly rasters of A0, A1 and ph1, were calculated per each year in the period 2007–2016, an average was calculated across the 10 years.

The seven variables included in this study were:

- Standard deviation of altitude (AltSd)
- Mean daytime Land Surface Temperature in °C (LstdMn)
- Mean amplitude of annual cycle of LST in °C (LstdAmp1)
- Peak timing of the annual cycle of LST, i.e. the day when the first harmonic of the temperature series reaches its maximum value (LstdDPk1)
- Mean greenness value (NDVIMn)
- Mean amplitude of annual cycle of greenness values (NDVIAmp1)
- Daily mean amount of rainfall in millimetres (RainMn).

### Data sources, collection and manipulation

Elevation was derived from the 20 m Digital Elevation Model of Italy (http://www.pcn.minambiente.it accessed 2^nd^ March 2018). The standard deviation (**AltSd)** of the values falling in the pixel at the reference scale (see next paragraph for details) was calculated. The standard deviation of altitude points out the local variability of the altimetry and resulted more informative than the mean altitude that in Italy is highly correlated with mean temperature (Pearson correlation coefficient = 0.85, p-value <0.01).

From the product MOD11A2 (MODIS/Terra Land Surface Temperature and Emissivity 8-Day L3 Global 1km Version-5), the daytime LST images (LSTD) were downloaded for ten complete years, from 01.01.2007 to 12.31.2016 [43]. Each raster was converted into WGS84–UTM33 coordinate system and the values into degree Celsius (°C). The four tiles covering Italy were mosaicked, and single raster files per date were archived, resulting in 46 images per year.

Linear temporal interpolation, spatial interpolation and climatology procedures [44] were run to fill missing pixel data. The dataset was then submitted to a Seasonal Trend Analysis to perform a harmonic regression on yearly data. The average over the decade of the yearly harmonics 0 and 1 were retained: amplitude 0 and amplitude 1 were named **LstdMn** and **LstdAmp1,** respectively. To facilitate interpretation, phase of the first harmonic was accounted as **LstdDPk1**: it is the day when the first harmonic of the temperature series reaches its maximum value and it was calculated applying the following formula

LstdDPk1=MOD(450‐LSTD_ph1,360)

where *MOD(n*, d) = n—d*INT(n/d), *MOD* function returns the remainder of two numbers after division. *LSTD_ph1* is the phase of the first harmonic of temperature.

*n* = number, is the number to be divided; *d* = divisor, is the number to divide with. The parameter *450* in formula is set to always have the peak in the positive x-axis.

The NDVI dataset was downloaded for the reference period from the product MOD13Q1 (MODIS/Terra Vegetation Indices 16-Day L3 Global 250m Grid SIN V006) [45], geographically processed and resulting in 23 images per year. Each yearly NDVI dataset was submitted to a Fourier decomposition as described for LST; the average over the decade of the yearly harmonics of amplitudes 0 and 1, were retained and named **NdviMn** and **NdviAmp1,** respectively. The phase of the harmonic 1 (representing the day of the year when the greenness reach its maximum value) is a variable highly dependent on the type of cultivation and averaging the values across the decade could mislead the ground truth. As the objective of this study is not to investigate the characteristics of field cultivation in Italy, we decide to not include it in the analyses.

Daily rainfall rasters for ten years, from 01.01.2007 to 12.31.2016, were downloaded from DEWETRA web application (http://dewetra.cimafoundation.org/dewetra/ accessed 2^nd^ March 2018): these rasters derive from ground weather stations managed by the Italian Civil Protection, interpolated trough the GRISO method at a 0.02 degrees [46]. The daily mean amount of rainfall was considered as variable (**RainMn**), being representative of the total rainfall of the year [47].

Pixels corresponding to lakes and cities were set to NoData in all dataset to avoid spurious and artificial effects of reflectance wavelength on these surfaces.

The geographical manipulation was performed in ArcMap 10.3.1 ESRI with routines in Python language; the Fourier analyses in Idrisi Taiga software and with Idrisi Macro Language. Routines in R software (https://www.r-project.org/ accessed 2^nd^ March 2018) and the Modis Reprojection Tool (https://lpdaac.usgs.gov/tools/modis_reprojection_tool accessed 2^nd^ March 2018) were used to download and process the MODIS images. The processing archive size and number of files is reported in S1 Table.

The spatial resolutions for our analyses were: i) 2 km (driven by DEWETRA rainfall data spatial resolution); ii) 1 km (driven by LSTD data spatial resolution) and iii) 250 m (driven by NDVI spatial resolution). From the original resolution of each dataset, an aggregation was performed to obtain the datasets at the three resolutions (2 km, 1 km and 250 m). The S2 Table reports the aggregation methods.

### Statistical analysis

For each of the three spatial resolutions (2 km, 1 km and 250 m), the following analyses were run.

All variables were standardized to a mean of zero and a standard deviation of one. A Principal Component Analysis (PCA) with a varimax rotation used to make the factors orthogonal and more interpretable, was first performed to take into account the correlation among the variables. The first three principal components were used as the axes for the environmental data space and the basis for the ecoregionalization and their scores were scaled in a 0–255 range for an RGB colour triplet representation [48,49]. All the values in the 10^th^ percentile were assigned to zero; all the values higher than the 90^th^ percentile were set to 255, linearly scaling all the others. In this way we mapped the first, second, and third principal component scores of each pixel to a red–green–blue (RGB) colour triplet. Pixels containing similar climatic and environmental combinations are coloured similarly.

The three components were then processed in a multivariate geographic k-medoids cluster analysis, a classical partitioning method that aggregates the dataset of *n* objects into *k* clusters known *a priori* [50]. The partition algorithm around medoids minimizes the average quadratic error between all points and the point centre of the cluster. The ‘optimal’ number of clusters was identified using the Silhouette technique on a priori groupings ranging between 10 and 50 [51]. This range was chosen to keep all landscape variability in Italy but avoiding a too fragmented clusterization.

Pixels belonging to the same cluster were displayed with the colour of the medoid of the corresponding cluster, thus colours reflect the distance in terms of information: similar colours reflect smaller distances between the cluster medoids, i.e. the pixels have similar environmental characteristics.

Once the ecoregionalization process was run at each spatial resolution, the three outputs were compared; for each pairwise combinations of resolutions *i* and *j* (250 m vs 1 km, 250 m vs 2 km, 1 km vs 2 km), we calculated:

- a confusion matrix with the percentage of the area that migrated from each cluster of resolution *i* to all clusters of resolution *j*;
- a confusion matrix with the Euclidean distance among the medoids in the 7-dimension space of the normalised variables to define the environmental similarity between each cluster of resolution *i* and clusters of resolution *j*.

This process provides a measure of the agreement between resolutions *i* and *j*.

All the statistical analyses were performed in R software version 3.3.2 (R Core team, https://www.r-project.org/) using *rasterVis* [52], *sp* [53] and *raster* packages for spatial analysis and visualization, *fpc* package [54] for cluster analysis, *rgl* package [55] and *vioplot* [56] for clusters representation.

### Entomological and disease case studies

To discuss the possible use of the ecoregionalization process, two case studies were investigated verifying the overlapping between specific ecoregions (output result at 250 m resolution) and the spatial distribution of *C*. *imicola* and *C*. *obsoletus/scoticus* bluetongue vector and West Nile disease outbreaks. Although the WNV has a complex life cycle, involving several bird species as reservoir hosts, in Italy the West Nile virus circulation and transmission are indisputably closely linked to the presence and distribution of its main vector, *Culex pipiens s*.*l*. [57,58].

#### BT vectors dataset

An Entomological Surveillance National Plan for bluetongue is in place since 2000 in Italy [59], stating field protocols for the collection of *Culicoides* and laboratory protocols for insects identification [60]. Information on single night catches is collected in a centralised database, from which presence/absence and abundance of *Culicoides* species incriminated as BTV vectors were derived: *C*. *imicola* and *C*. *obsoletus/scoticus* (this taxon including the two cryptic species *C*. *obsoletus* and *C*. *scoticus*) [61,62]. Collections performed during winter period (December-March), with a number of insects lower than 5 per night and with no specimens belonging to one of the considered vector species were removed, and only correctly georeferenced sites were kept for the analysis. From the original 150,000 collections from August 2000 to August 2017, those analysed for *C*. *imicola* were 73,182, located in 3,630 sites across Italy; 6,898 collections were analysed in 1,511 sites for *C*. *obsoletus/scoticus*. For each cluster, the percentage of positive sites (a positive site is defined as a site with at least one specimen of the considered vector species collected at least in one night) and the average abundance in positive sites (through the whole period considered), were calculated. Since the number of sites was variable in the clusters, the uncertainty of the percentage was calculated using a Beta distribution and its 95% confidence intervals.

#### WN outbreaks dataset

The re-occurrence of WND in Italy in 2008 heavily affected the Po river delta in Northern Italy, leading to the establishment of an integrated national surveillance plan that combines human cases reported by local health authorities and animal surveillance data [63–65]. Besides, extensive entomological regional surveillance plans were put in place in the endemic areas of Northern Italy, nevertheless the entomological data are not available at national level. As reported by Calzolari *et al*. [57], in the endemic area of Pianura Padana, a robust entomological monitoring allowed the early detection of WNV in *Culex pipiens s*.*l*. pools before the occurrence of human cases. Thus we can assume that outbreaks locations represent a good proxy for *Culex pipiens s*.*l*. presence being this vector the species most involved in the WNV circulation in Italy [57,58].

WND outbreaks reported in Italy in the frame of the animal surveillance were then investigated to verify consistency with ecoregions. No human cases were included for privacy reason, but this does not affect the results as their distribution is within the geographical distribution of animal cases [64].

WN virus detection and seroconversions in horses and birds, equine clinical cases and positive entomological collections were derived from the Italian National Surveillance Information System (https://www.vetinfo.sanita.it/ accessed 2nd March 2018) collecting the notification of animal disease outbreaks in Italy [66].

## Results

### Ecoregionalization

**Fig 1:**
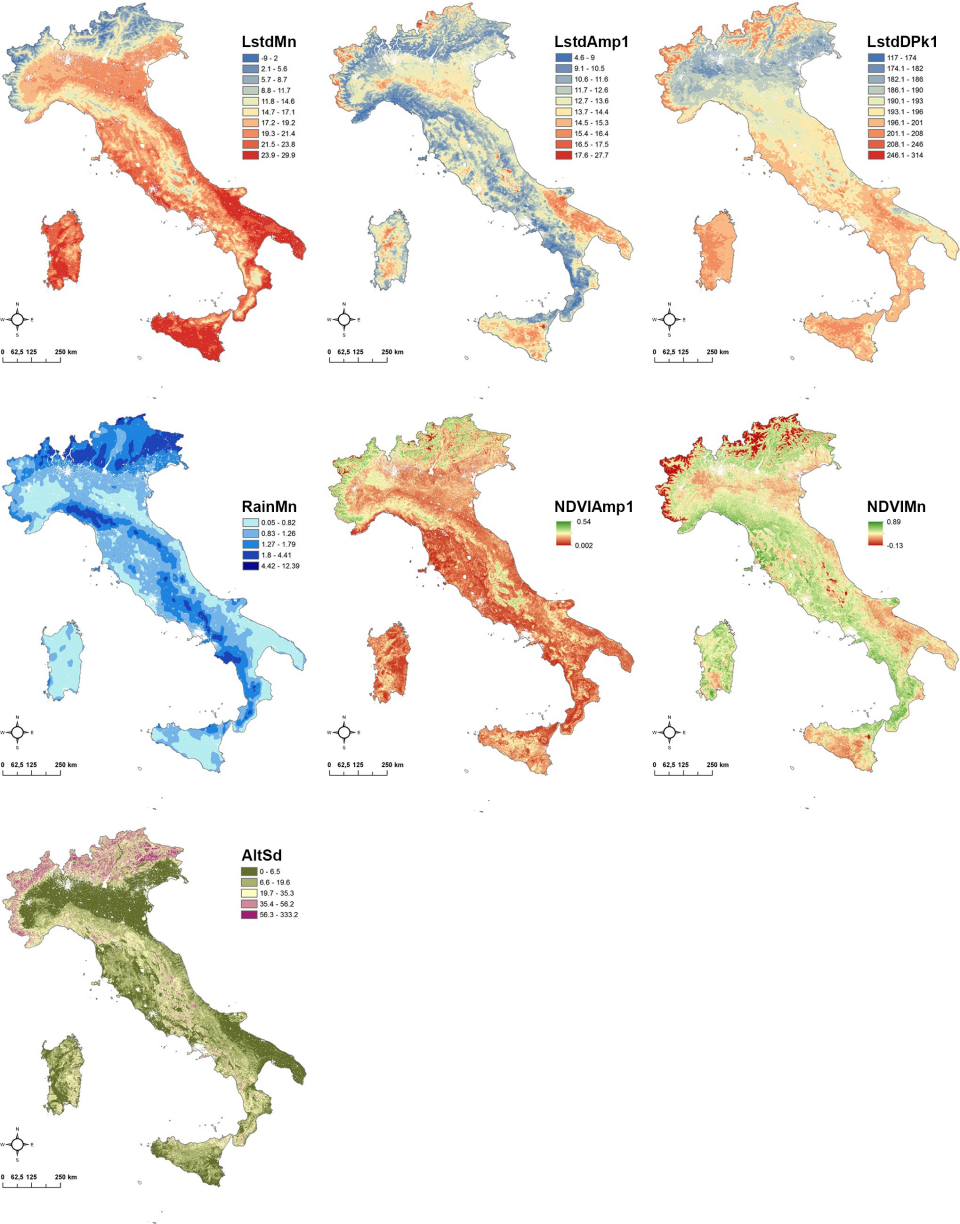
Raster input variables. The input variables are: mean daytime land surface temperature (LstdMn), mean amplitude of annual cycle of land surface temperature (LstdAmp1), peak timing of annual cycle of land surface temperature (LstdDPk1), daily mean amount of rainfall (RainMn), mean amplitude of annual cycle of greenness values (NDVIAmp1), mean greenness value (NDVIMn), standard deviation of altitude (AltSd).

Fig 1 shows the seven environmental and climatic input variables processed for PCA. The S1 Fig reports the correlation matrix among the seven variables.

The varimax rotation matrix highlights the relations between the input variables and the factors derived from PCA (Table 1 for 250 m resolution, S3 and S4 Tables for 1 km and 2 km respectively). The proportion of variance of the first three components was 79.35% at 250 m, 80.06% at 1 km, and 79.78% at 2 km spatial resolutions.

**Table 1:** Relationship between the input variables and the factors of the PCA at 250 m spatial resolution.

	PC1 (blue)	PC2 (green)	PC3 (red)	PC4	PC5	PC6	PC7
**LstdMn**	0.53	-0.17	0.04	-0.22	0.23	-0.27	0.72
**LstdAmp1**	0.35	0.52	-0.14	0.07	-0.07	-0.68	-0.34
**LstdDPk1**	0.05	0.33	0.83	-0.15	0.38	0.12	-0.13
**NDVIMn**	-0.01	-0.67	0.16	-0.42	0.04	-0.38	-0.44
**NDVIAmp1**	-0.34	0.35	-0.35	-0.76	0.23	0.03	0.06
**RainMn**	-0.49	-0.08	-0.08	0.41	0.64	-0.39	0.12
**AltSd**	-0.48	0.08	0.36	-0.08	-0.58	-0.39	0.37
***Cumulative proportion of variance***	***0***.***39***	***0***.***64***	***0***.***79***	***0***.***87***	***0***.***93***	***0***.***97***	***1***.***00***

**Fig 2:**
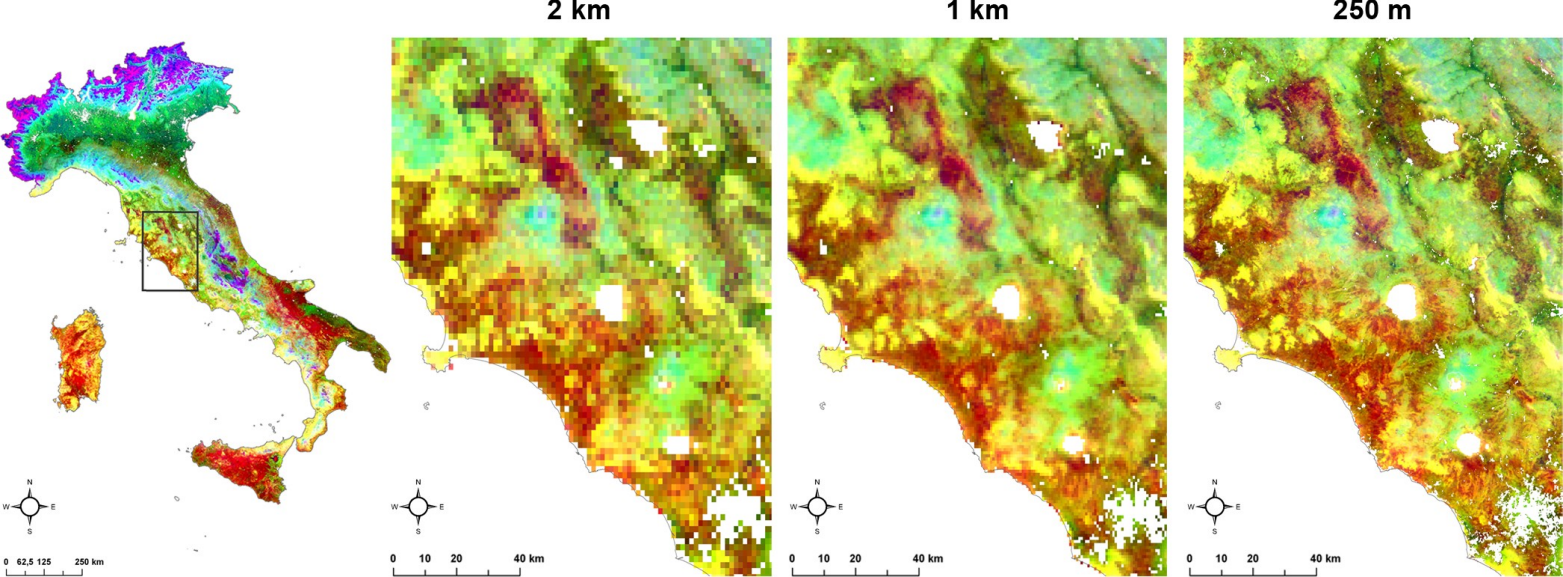
RGB classification: Pixels similar in terms of climate and environment are similar in colours. The three spatial resolutions 2 km, 1 km, 250 m are reported from left to right for a zoomed area (black box on the left map of Italy).

The first three factors of the PCA were associated to the colour triplet Blue-Green-Red: PC1 “Mountains”, which was mainly driven by steep slopes, low mean temperature, high precipitations, was associated to the Blue colour in reverse mode (i.e. light blue is associated to higher values of PC1, darker blue goes to pixels with low PC1); PC2 “Vegetation greenness”, characterised by low seasonal temperature variation and high vegetation presence, was associated to the Green colour in reverse mode; PC3 “Length of hot season”, characterised by a long hot season was associated to the Red colour (S2 Fig). When applying this RGB colour scheme, the resulted map showed similar pixels in similar colours (Fig 2).

**Fig 3:**
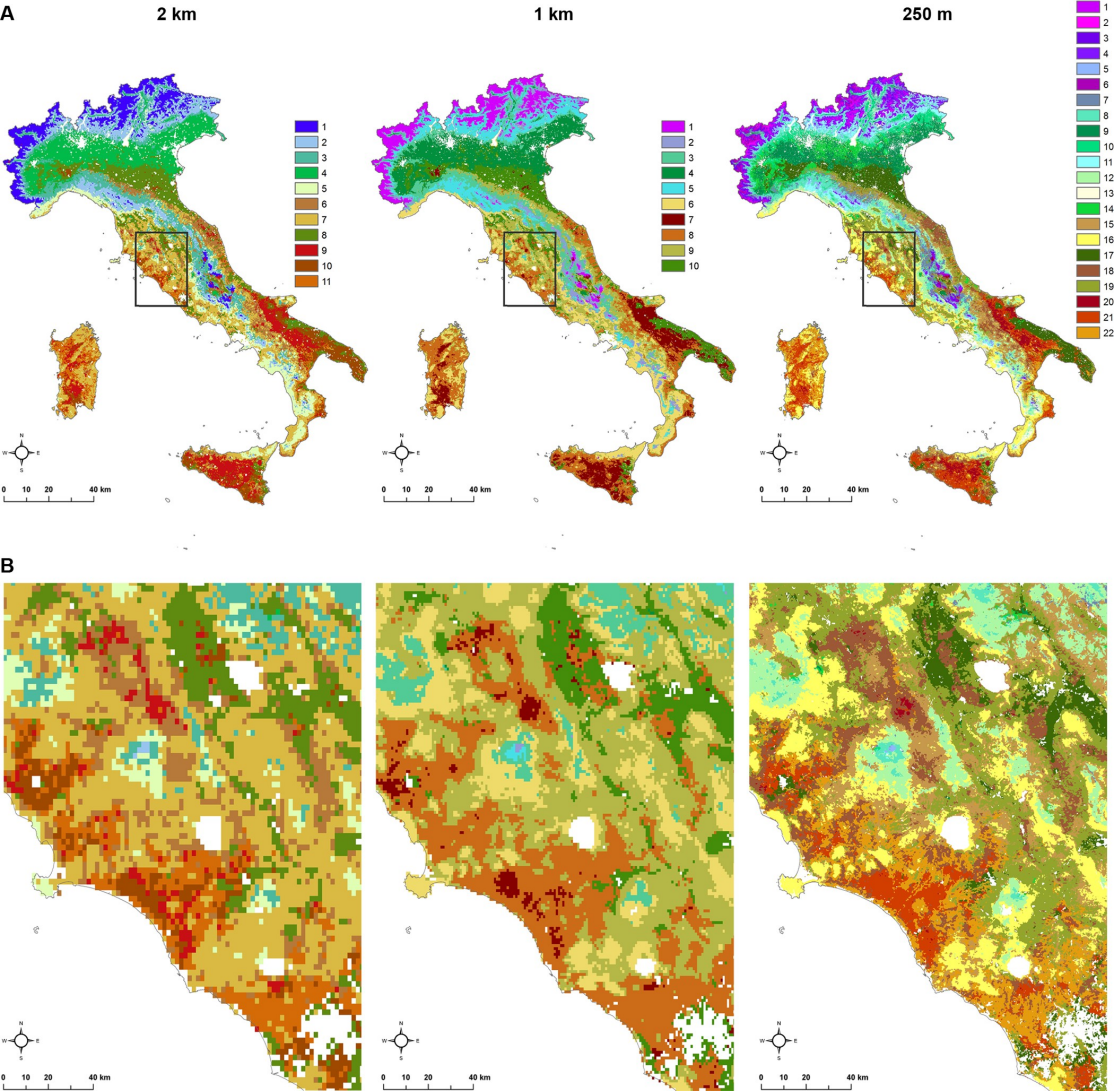
Cluster results at the spatial resolutions of 2 km, 1 km, 250 m from left to right, for whole Italy (A) and in a zoomed area (B).

The multivariate k-medoids geographic clustering, applied to the optimal number of clusters identified by the Silhouette (S3 Fig), produced 11 clusters at 2 km spatial resolution, 10 clusters at 1 km and 22 clusters at 250 m (Fig 3). The total number of pixels in each ecoregion map is 79,619 (2 km), 312,673 (1 km) 4,869,825 (250 m) respectively (S5 Table).

**Fig 4:**
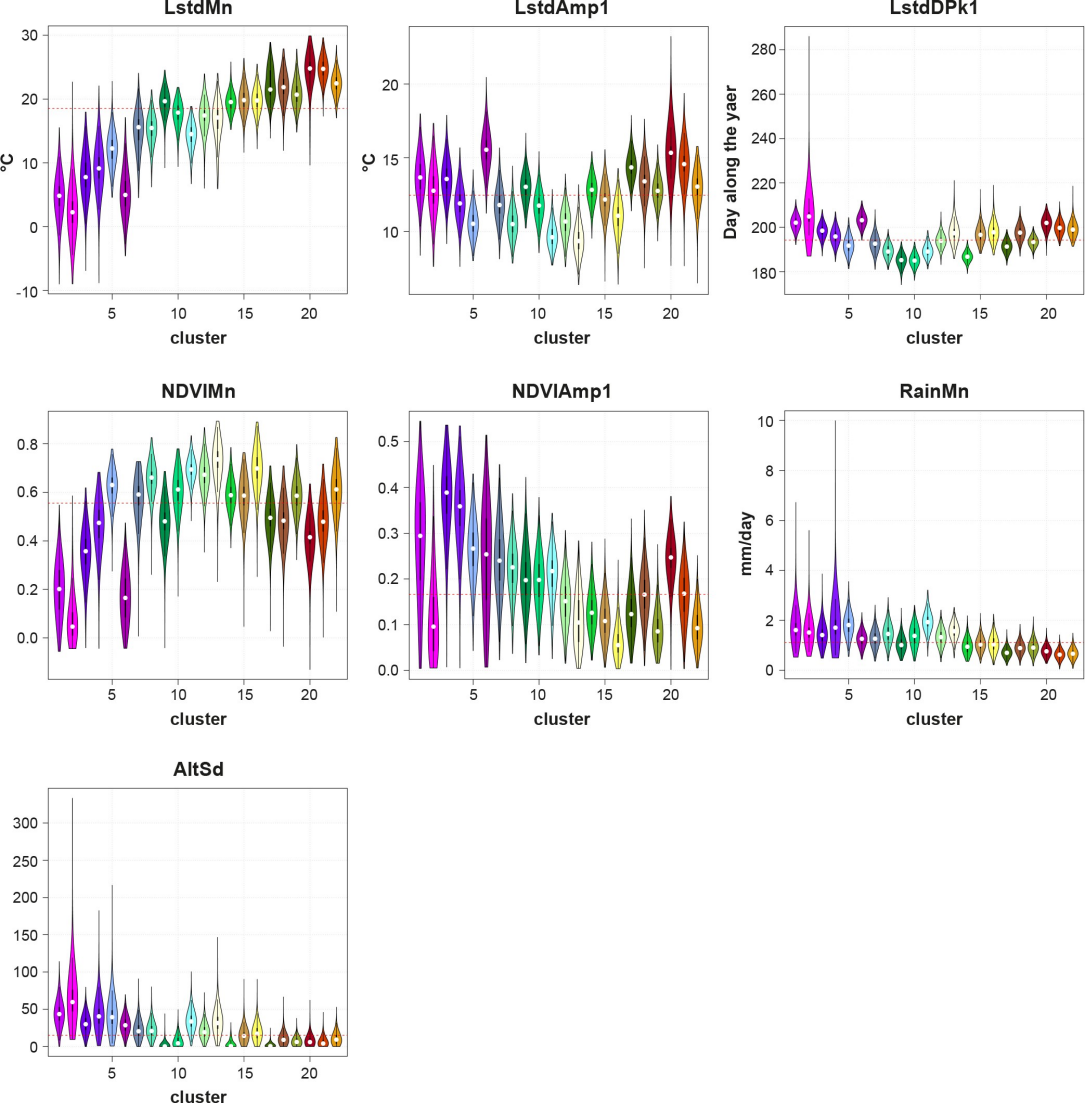
Violin plots depicting the characteristics of the seven input variables in the 22 ecoregions (250 m spatial resolution). The white marker is the median of the data, the box indicates the interquartile range, with a kernel density overlaid. The red line reports the average value across all clusters. Each violin has the same color of the corresponding ecoregion.

The main characteristics of each ecoregion are shown through violin plots of the input variables (Fig 4 for 250 m resolution; S4 and S5 Figs for 1 and 2 km spatial resolutions); the shape and the size of the violins describe the distribution of the values around the centroid, i.e. it is a quantitative measure of variability of each factor within an ecoregion.

At 250 m resolution, clusters representing mountainous areas (1–7) have a prevailing purple-pink colour with low mean temperature, above average seasonal variance in temperature, low average values of vegetation greenness and high annual variations in vegetation, with consistent rains and high local variance in elevation. The green-based clusters (approx. 8–17) have a common high value of average vegetation, with the variance across the year depending on the land cover (low variation for forests, high for cultivated fields). The differences among the clusters of this group are mainly related to topography, rain and temperature variation. The group of clusters (approx. 18–22) are red-based, highlighting their characteristics of general high mean temperature, high variance across the year and presence of long hot season, low rainfall rates and flat areas. The clusters are differentiated by distinct crop types reflecting in different amount and seasonality of vegetation indices.

**Fig 5:**
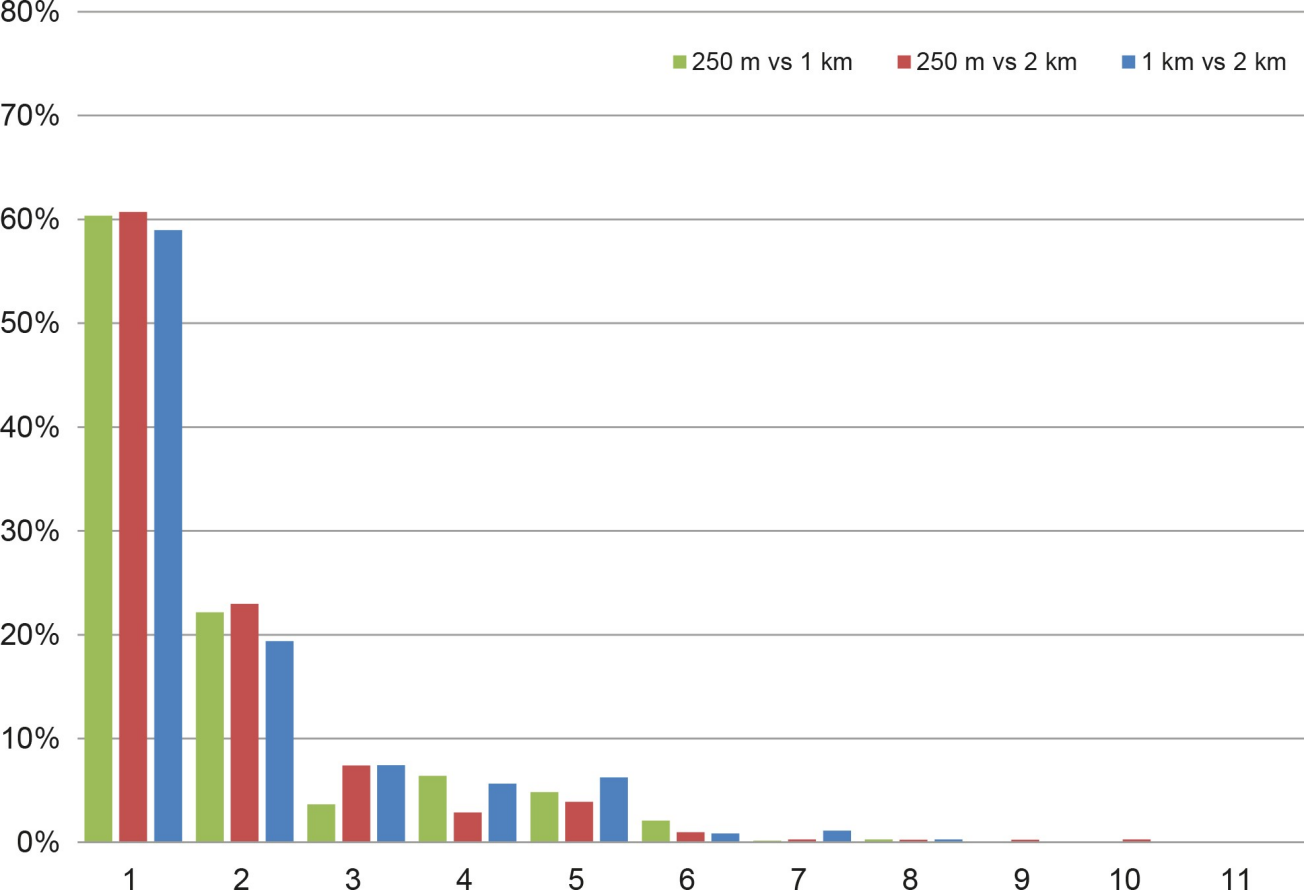
Migration area of the pixels from the resolution *i* to the more similar clusters in resolution *j*. The three pairwise comparisons show that the 80% of the area of a classification migrated to the most similar and to the second most similar clusters of the other classification.

The robustness of the three output results was evaluated through the total area migrating from one cluster at resolution *i* to the more similar ones in resolution *j*. The three pairwise comparisons show that the 80% of the area of a classification migrated to the most similar and to the second most similar clusters of the other classification (Fig 5).

### Entomological and disease case studies

**Fig 6:**
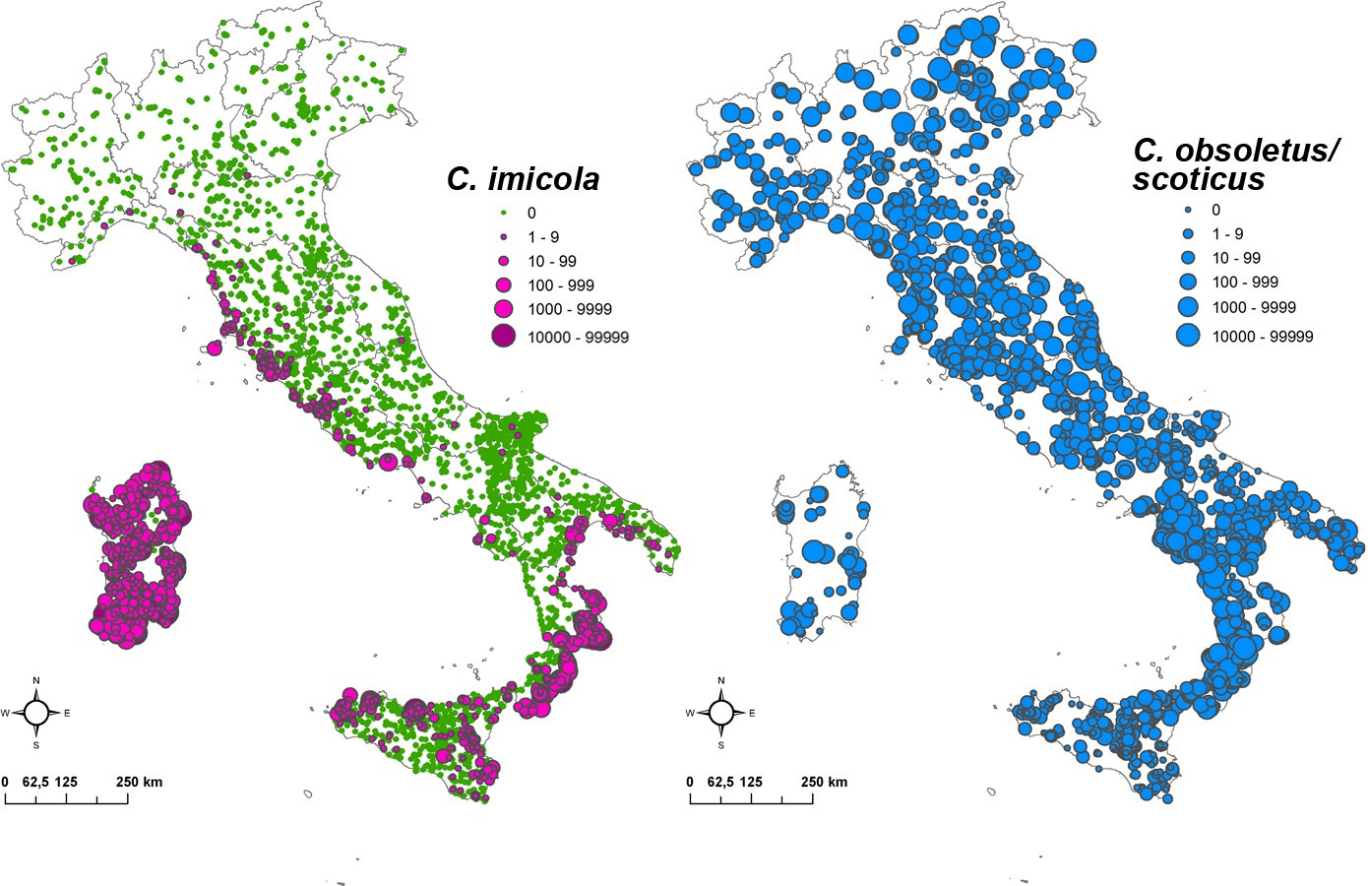
Spatial distribution of *C*. *imicola*, *C*. *obsoletus/scoticus* in Italy. The maximum specimens catched in the most favourable period (April–November) in the period August 2000- August 2017.

As Fig 6 shows, *C*. *imicola* distribution is clustered in specific areas of Southern Italy. Conversely, *Culicoides obsoletus/scoticus* is widespread across the country. The number of trapping sites and collections performed per cluster is reported in Table 2. Ecoregions 1, 2 and 6 had no trapping site as they represent mountain areas with no (or few) livestock.

**Table 2:** Number of trapping sites and collections per ecoregion for *Culicoides imicola*, *C*. *obsoletus/scoticus*.

ecoregion	*C*. *imicola* dataset		*C*. *obsoletus/scoticus* dataset	
	Number of sites	Number of collections	Number of sites	Number of collections
**1**	0	0	0	0
**2**	0	0	0	0
**3**	10	643	3	16
**4**	3	9	2	2
**5**	12	373	5	16
**6**	0	0	0	0
**7**	47	1511	23	70
**8**	48	2717	32	137
**9**	100	6817	55	638
**10**	68	5244	48	382
**11**	5	9	2	2
**12**	151	2373	75	191
**13**	26	133	15	37
**14**	76	4756	46	395
**15**	422	6228	180	831
**16**	307	3549	162	369
**17**	320	10372	148	877
**18**	376	4963	139	955
**19**	484	8693	194	733
**20**	150	1561	52	156
**21**	684	7974	213	602
**22**	341	5257	117	489
**TOTAL**	**3,630**	**73,182**	**1,511**	**6,898**

**Fig 7:**
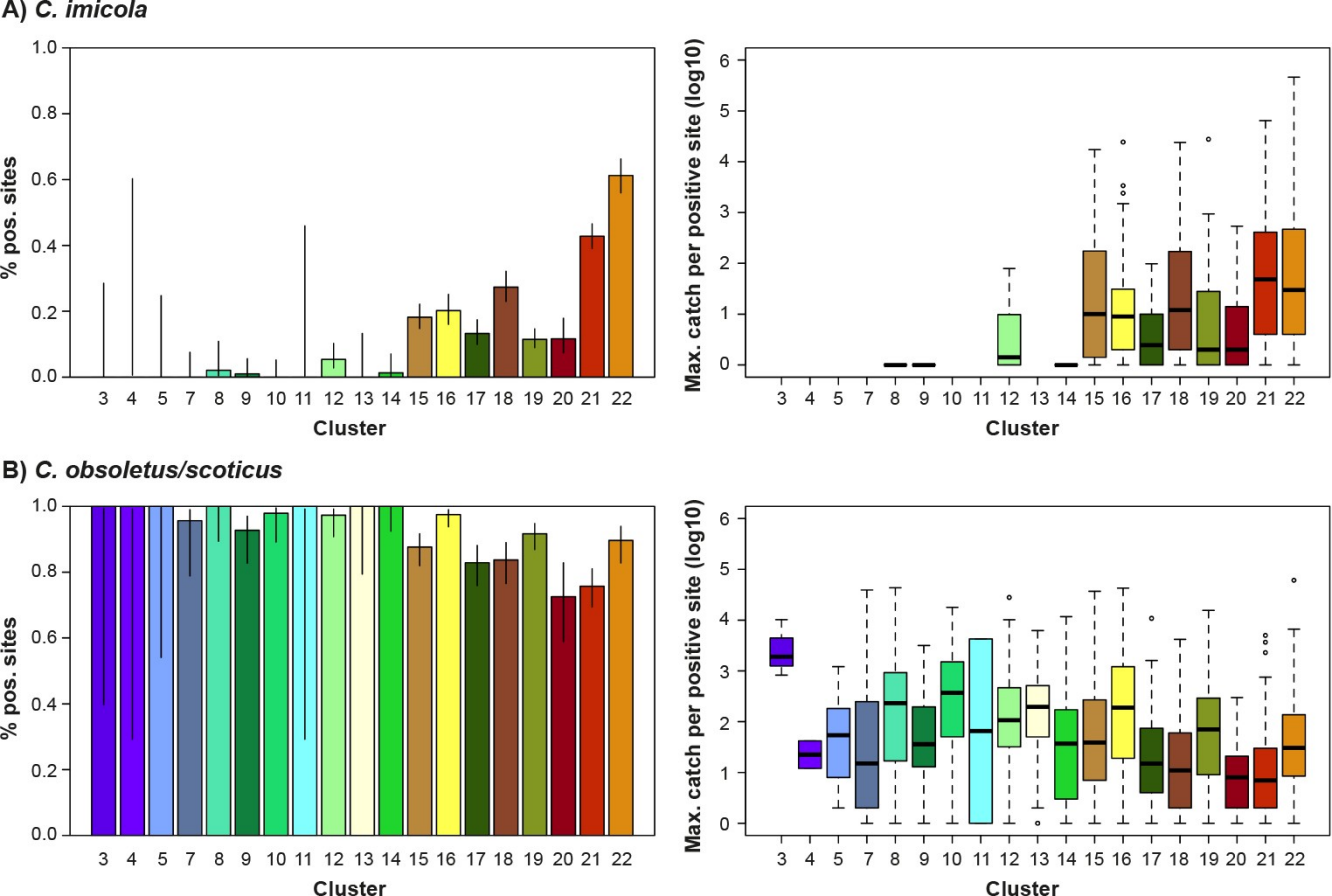
Distribution of *C*. *imicola* (A) and *C*. *obsoletus/scoticus* (B) in the 22 Italian ecoregions, as quantified by the proportion of positive sites by cluster (left) and the maximum catches per positive site (right).

Fig 7 shows the vector taxa as quantified by the proportion of positive sites (sites in which the species was present) by cluster (left) and the maximum number of collected midges in one night collection (i.e. maximum number of specimens) per positive site (right). The figure shows how *C*. *imicola* is mostly distributed within the two reddish ecoregions 21 and 22, where the percentage of positive sites is 43% (95% confidence interval 39%-47%) and 61% (95% confidence interval 56%-66%) respectively. When considering only the positive *C*. *imicola* sites (n = 851), the majority of sites are either located in ecoregions 21 and 22 (487 presence sites) or within 2 km from these two ones (278 positive sites).

*C*. *obsoletus/scoticus*, are widespread among all the ecoregions identified, showing adaptability to a wide range of climatic and environmental conditions: for *C*. *obsoletus/scoticus* the percentage of positive sites ranged from 71% to 100% in all the clusters, although some of them (i.e. clusters 3, 4, 5, 11) have only few sites as proven by the wide confidence interval bar.

**Fig 8:**
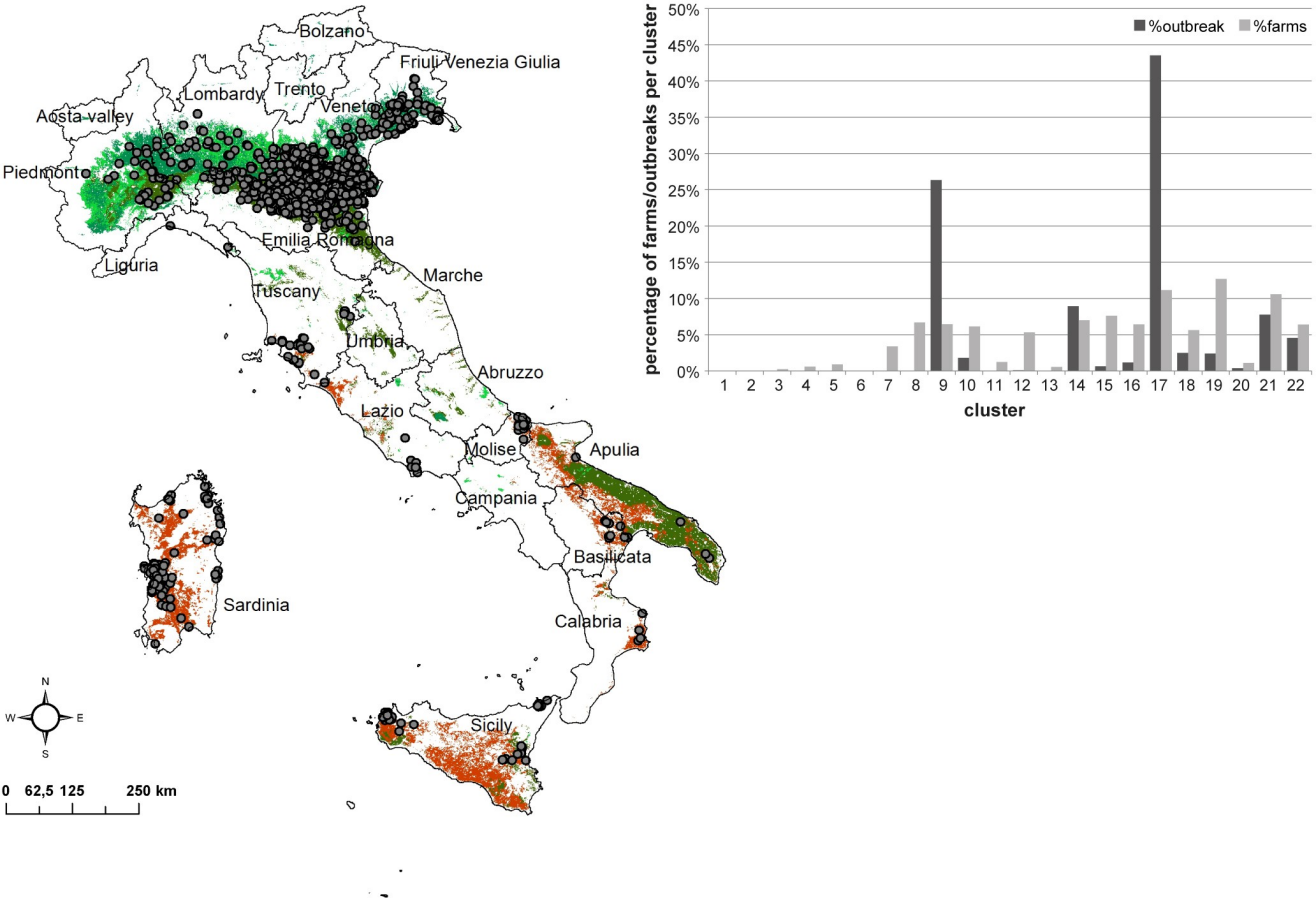
Spatial distribution of 2008–2016 WND outbreaks reported in Italy in the frame of animal surveillance overlaid to the clusters 9, 14, 17 and 21 where the 87% of outbreaks was located (map). In the graph, the percentage of outbreaks (in dark grey) and equine farms (in light grey) per ecoregion are reported.

Fig 8 reports the spatial distribution of 1,259 WND outbreaks notified in the frame of the animal surveillance in Italy in the years 2008–2016, overlayed to the ecoregions 9, 14, 17 and 21. Despite the even distribution of equine farms across the country, the 87% of outbreaks are located in the four mentioned ecoregions.

## Discussion

In this paper, a multivariate statistical clustering algorithm was applied to seven climatic and environmental variables to classify the Italian territory in similar regions at three spatial resolutions, 250 meters, 1 km and 2 km delineating 22, 10 and 11 ecoregions, respectively.

The coarser ecoregion division (2 km pixel resolution) accurately captures intuitively understood environmental differences in Italy. As in the most recent hierarchical classification available [2], although not properly comparable because of the differences in data and approach applied, the map distinguishes the high mountains of Alps (cluster 1), the pre-Alps and Apennines chain (clusters 2 and 3), the flat and cultivated areas of Po valley and of part of South peninsula (clusters 4 and 8), the hilly rainy areas on the Tyrrhenian coast with stable vegetation along the year (clusters 5 and 7), the pre-Apennines hills (most of the territory below 600 m a.s.l.) (cluster 6), the very hot areas of southern Italy and islands without rain with little vegetation fluctuating during the year (cluster 9) and very flat (cluster 10), the undulating very hot landscapes (cluster 11). This coarser classification is consistent with the one at 1 km resolution which defined 10 ecoregions. The number of classes increases to 22 at 250 m pixel resolution, giving further details nested in the broader patterns of the previous two classifications.

Rather than rely on expertise or follow a hierarchical deductive method [2], the process reads the environmental information in the 7-space data, and statistically groups them, assigning a colour to each group: similar colours (in the RGB palette) mean similar environment. The top of Alps in northern Italy and the top of Apennine chain are more purple/blue-related (Blue = “mountains”), and they are characterized by steep slopes, low mean temperature and high precipitations. Southern Italy, Sardinia and Sicily islands are more reddish (Red = “Length of hot season”) being dominated by long hot season, with a mix of green (Green = “Vegetation greenness”), so getting to orange-brown. Green areas are related to high vegetation (either as cultivated areas or natural landscapes) and to moderate seasonal temperature variation, the latter mitigated by the presence of vegetation leaves.

Pixels with similar environments should be classified in the same ecoregion even if widely separated geographically [67]. For this reason, the geographical position of the pixels and the geographical neighbourhood were deliberately taken out from the clustering process so to identify clusters also spatially disjoint. Notwithstanding, ecoregions tend to be geographically cohesive because of the spatial autocorrelation usually present in the environmental data [48]. There is generally a gradient in colour among close pixels, highlighting similarity in the environmental conditions and information consistency, and when present, the salt and pepper effect is mainly due to the typical fragmented land use of Italian territory.

The ecoregions maps show a geographical coherence at the three spatial resolutions (250 m, 1 km, 2 km): the 80% of the pixels of a resolution migrated to the most similar and to the second most similar cluster of the other resolutions. This means that moving from a coarser to a finer spatial resolution (or vice-versa), the area covered by the corresponding pixels is classified similarly (in terms of colours, i.e. of eco-climatic characteristics). The choice of the resolution to be selected/to be produced depends mainly on the final use of the classification.

To our knowledge, this is the first attempt at producing a quantitative data-driven approach to delineate eco-climatic areas in Italy using variables associated to VBDs and VBDs vectors. The idea arises from the potential application of such a layer in support to surveillance and control management.

It is reasonable to assume a more probable spread of a VBD within each ecoregion, given the similar characteristics, rather than between different clusters. This is a first useful information from a public health perspective. Pixels of the same colour belong to the same cluster, and therefore have, on average, the same environmental characteristics. The WND case study highlights a strong correspondence between the location of WND cases and few greenish ecoregions (9, 14, 17 and 21 at 250 m spatial resolution). Since 2008, WND strongly hit Northern Italy and in particular the Po Valley [68]. This intensively cultivated area is characterized by the presence of rivers and wetlands where ornithophilic mosquitoes and migratory birds are in close contact, allowing the transmission of WNV and the establishment of enzootic cycle of infection. The natural areas as well as the agricultural and urban areas, characterized by the constant presence of stagnant water provide potential breeding habitats for different mosquito species, including *Culex pipiens* s.l., considered the main vector of WNV, and of other *Flavivirus* such as USUTU virus, in the whole Italian Peninsula [57,58]. Although 80% of the WND outbreaks fall in the greener 3 ecoregions mentioned, the 7% are found in reddish cluster 21 representing risk areas in the southern part of Italy. The low percentage of cases in these areas and their clustered distribution inside the ecoregion, can be explained by the local presence of specific factors, additional to the ones included in the present ecoregion definition, such as humid areas and migratory bird settlements (e.g. ponds in Oristano province in Western Sardinia; Lentini Lake in Eastern Sicily, Stagnone Lagoon and Marsala salt ponds in Western Sicily).

Second, the spread of disease from one part of Italy to another geographically distant, but belonging to the same ecoregion, would be an important risk factor to take into account for disease control measures. The spatial structure of the ecoregions in relation to known infections could therefore be used to prioritize animal movement control such as to prevent infections from one geographic part of an ecoregion to another.

Third, this approach could also inform entomological surveillance strategies identifying similar places far apart in which to intensify or reduce the sampling activity. In the case of *C*. *imicola* in Italy, this BT vector revealed a strong association with two ecoregions, the 21 and 22. *Culicoides imicola* has already been proved to be associated with specific habitat characterised by high mean temperatures, dry environments, flatten and sunny surfaces, low vegetation [17,69,70] and in Italy the combination of these favourable factors clearly delineates the two specific ecoregions. These two ecoregions are mainly located in Sardinia, Sicily, Ionian part of toe of Italy, Tyrrhenian coast of Lazio and Tuscany, where the distribution of *C*. *imicola* is documented. The ecoregion map here produced highlights that these clusters (at risk) are also present in river valleys of Adriatic coast where few or none cases were reported, and in the area laying between Apulia and Basilicata regions; a deeper entomological investigation might verify if and which local conditions may locally decrease the vector population (type of crops, farming types, absence of water for larval sites, etc.).

If the approach captures the environmental preferences of *Culicoides imicola*, whose distribution in Italy is spatially constrained, it seems less accurate with the *Culicoides Obsoletus/scoticus*, a species with a broader distribution across the country. *Culicoides obsoletus/scoticus* is a taxon not showing evidence of prevalence in specific ecoregions, in agreement with previous studies [62,71]. In terms of abundances, the highest values were found in ecoregions characterized by high vegetation indices, but with different seasonal patterns. The two cryptic species *C*. *obsoletus* and *C*. *scoticus* are both related to vegetation, but with *C*. *scoticus* associated to more natural environments and *C*. *obsoletus* being a more widespread species, including urban areas [25,62]. *Culicoides obsoletus* in Italy is largely the most abundant of the two [62,72], thus it might have played a major role in the even distribution of positive sites. A more detailed set of variables [25] could derive a tailored ecoregionalization for these two species.

In general terms, the availability of specific environmental datasets is a key point in the development of such an approach. Some variables such as soil composition, permeability and capacity of retain water are key factors in defining vector habitat [71], but detailed information covering the entire Italian territory is not available and the ecoregionalization might be lacking in considering this aspect.

An additional consideration is the role of hosts in defining ecoregions. This aspect, deliberately taken out from our generic classification, it is important when tailoring the method to a specific pathogen/disease because the ecoregions will be based not only on climatic and environmental factors (suitability for the vector), but also on the availability of hosts (suitability for the disease).

### Future perspectives

The multivariate clustering method can be extended to a geospatiotemporal clustering in which multi-dimensional datasets of temporal variables would be used for describing and tracking the similarity of ecosystem properties through time, either retrospective or forward. This could be used to detect changes in landscape features that may underline the areas of emergence of new diseases, the resurgence of old ones, or changes in transmission opportunities of established vector-borne pathogens [12].

Applied in Italy on weekly or monthly basis, a “dynamic ecoregion” definition would help to define which areas are similar in which period, and it might represent an added value in case of virus entrance in a specific time of the year so to adopt priority intervention and control measure in similar areas at higher risk. Moreover the method could be replicated including datasets on present and future climatic conditions, to investigate the evolution of ecoregions in Italy under climate change scenarios [21,26].

Although it still remains a rich and complex process requiring different expertise, ecoregionalization could contribute in the development of accurate early warning systems so to timely prevent and control VBDs spread through a harmonized and targeted surveillance systems, in space and time, for host and vectors.

## Supporting information

S1 Table Number of images processed and archive size of the climatic and environmental products. (DOCX)

S2 Table Aggregation methods of the original dataset to obtain the resolutions (2 km, 1 km and 250 m) of the factors used in the statistical analysis. (DOCX)

S3 Table Relationship between the input variables and the factors of the Principal Component Analysis at 1 km spatial resolution. The first factor of PCA is then associated to the Blue colour channel in reverse mode, the second component is associated to Green in reverse mode, the third component PC3 to the Red.
(DOCX)

S4 Table Relationship between the input variables and the factors of the Principal Component Analysis at 2 km spatial resolution. The first factor of PCA is then associated to the Blue colour channel in reverse mode, the second component is associated to Green in reverse mode, the third component PC3 to the Red.
(DOCX)

S5 Table Number of pixels in each ecoregion map, divided by cluster. (DOCX)

S1 Fig Correlation matrix among the seven variables at 2 km spatial resolution. (TIF)

S2 Fig Association between PCA components and RGB values at 250 m spatial resolution. (TIF)

S3 Fig The optimal number of clusters for each spatial resolution identified by the Silhouette technique: 11 clusters at 2 km, 10 clusters at 1 km and 22 clusters at 250 m spatial resolution. (TIF)

S4 Fig Violin plots depicting the characteristics of the seven input variables in the 10 ecoregions (1 km spatial resolution). The violin plots show the probability density of the data at different values; they include a marker (white point) for the median of the data and a box indicating the interquartile range, with a kernel density overlaid. The red line reports the average value across all clusters. Each violin has the same color of the corresponding ecoregion.
(TIF)

S5 Fig Violin plots depicting the characteristics of the seven input variables in the 11 ecoregions (2 km spatial resolution). The violin plots show the probability density of the data at different values; they include a marker (white point) for the median of the data and a box indicating the interquartile range, with a kernel density overlaid. The red line reports the average value across all clusters. Each violin has the same color of the corresponding ecoregion.
(TIF)

S1 File Structure of entomological and farm data. (XLSX)

S2 File Raster data of ecoregion map with 22 classes at 250 meters spatial resolution in geotiff format. (7Z)
