# Supplementary material for: Defining ecological regions in Italy based on a multivariate clustering approach: A first step towards a targeted vector borne disease surveillance
Source: PLoS One. 2019 Jul 3;14(7):e0219072. doi: 10.1371/journal.pone.0219072 (PMC6608978; doi:10.1371/journal.pone.0219072)
Supplement: S1 Table — (DOCX) [file pone.0219072.s001.docx]

S1 Table. Number of images processed and archive size of the climatic and environmental products.

| Year | Number of images/size | | |
| --- | --- | --- | --- |
|  | RAIN DEWETRA | LSTD MOD11A2 | NDVI MOD13Q1 |
| Spatial resolution | 2 km | 1 km | 250 m |
| Temporal resolution | 1 day | 8 days | 16 days |
| 2007 | 365 / 114 MB | 46 / 391 MB | 23 / 4.38 Gb |
| 2008 | 366 / 115 MB | 46 / 391 MB | 23 / 4.37 Gb |
| 2009 | 365 / 114 MB | 46 / 391 MB | 23 / 4.37 Gb |
| 2010 | 365 / 114 MB | 46 / 389 MB | 23 / 4.38 Gb |
| 2011 | 365 / 114 MB | 46 / 389 MB | 23 / 4.39 Gb |
| 2012 | 366 / 115 MB | 46 / 399 MB | 23 / 4.40 Gb |
| 2013 | 365 / 114 MB | 46 / 556 MB | 23 / 7.68 Gb |
| 2014 | 365 / 114 MB | 46 / 553 MB | 23 / 7.67 Gb |
| 2015 | 365 / 114 MB | 46 / 567 MB | 23 / 7.70 Gb |
| 2016 | 366 / 115 MB | 46 / 550 MB | 23 / 7.67 Gb |
| **total** | **3653 / 1.10 Gb** | **460 / 4.48 Gb** | **230 / 57.1 Gb** |
