# Supplementary material for: Defining ecological regions in Italy based on a multivariate clustering approach: A first step towards a targeted vector borne disease surveillance
Source: PLoS One. 2019 Jul 3;14(7):e0219072. doi: 10.1371/journal.pone.0219072 (PMC6608978; doi:10.1371/journal.pone.0219072)
Supplement: S2 Table — (DOCX) [file pone.0219072.s002.docx]

S2 Table. Aggregation methods of the original dataset to obtain the resolutions (2 km, 1 km and 250 m) of the factors used in the statistical analysis.

|  |  | Input for the analysis at | | |
| --- | --- | --- | --- | --- |
|  | *Original resolution of the dataset* | 2 km | 1 km | 250 m |
| **AltSd - standard deviation of altitude** | *20 m* | Aggregation: standard deviation of the falling pixel values | Aggregation: standard deviation of the falling pixel values | Aggregation: standard deviation of the falling pixel values |
| **LstdMn, LstdAmp1, LstdDPk1 - daytime land surface temperature** | *1 Km* | Aggregation: mean of the falling pixel values | Original resolution | Repetition of original value is in the overlapping cells |
| **NDVIMn, NDVIAmp1 - greenness index** | *250 m* | Aggregation: mean of the falling pixel values | Aggregation: mean of the falling pixel values | Original resolution |
| **RainMn - daily mean amount of rainfall** | *0.02 °* | Resample | Repetition of original value is in the overlapping cells | Repetition of original value is in the overlapping cells |
