# Supplementary material for: Defining ecological regions in Italy based on a multivariate clustering approach: A first step towards a targeted vector borne disease surveillance
Source: PLoS One. 2019 Jul 3;14(7):e0219072. doi: 10.1371/journal.pone.0219072 (PMC6608978; doi:10.1371/journal.pone.0219072)
Supplement: S4 Table — The first factor of PCA is then associated to the Blue colour channel in reverse mode, the second component is associated to Green in reverse mode, the third component PC3 to the Red. (DOCX) [file pone.0219072.s004.docx]

S4 Table. Relationship between the input variables and the factors of the Principal Component Analysis at 2 km spatial resolution. The first factor of PCA is then associated to the Blue colour channel in reverse mode, the second component is associated to Green in reverse mode, the third component PC3 to the Red.

| 2 km | PC1 | PC2 | PC3 | PC4 | PC5 | PC6 | PC7 |
| --- | --- | --- | --- | --- | --- | --- | --- |
| LstdMn | 0.53 | -0.17 | 0.04 | -0,21 | 0,28 | 0,21 | 0,72 |
| LstdAmp1 | 0.35 | 0.53 | -0.15 | 0,06 | -0,04 | 0,70 | -0,29 |
| LstdDPk1 | 0.05 | 0.32 | 0.84 | -0,13 | 0,37 | -0,12 | -0,16 |
| NDVIMn | -0.01 | -0.68 | 0.15 | -0,42 | 0,06 | 0,41 | -0,42 |
| NDVIAmp1 | -0.35 | 0.35 | -0.35 | -0,75 | 0,26 | -0,06 | 0,05 |
| RainMn | -0.49 | -0.09 | -0.09 | 0,43 | 0,65 | 0,34 | 0,12 |
| AltSd | -0.49 | 0.08 | 0.35 | -0,09 | -0,53 | 0,41 | 0,42 |
| *proportion of variance* | *0.3972* | *0.2504* | *0.1502* | *0.0757* | *0.0587* | *0.0410* | *0.0269* |
| *Cumulative proportion of variance* | *0.3972* | *0.6476* | *0.7978* | *0,8734* | *0,9321* | *0,9731* | *1* |
