# Supplementary material for: Defining ecological regions in Italy based on a multivariate clustering approach: A first step towards a targeted vector borne disease surveillance
Source: PLoS One. 2019 Jul 3;14(7):e0219072. doi: 10.1371/journal.pone.0219072 (PMC6608978; doi:10.1371/journal.pone.0219072)
Supplement: S5 Table — (DOCX) [file pone.0219072.s005.docx]

**S5 Table. Number of pixels in each ecoregion map, divided by cluster.**

| **Cluster ID** | **Number of pixels per resolution** | | |
| --- | --- | --- | --- |
|  | **250 m** | **1 km** | **2 km** |
| 1 | 115,825 | 23,948 | 6,444 |
| 2 | 18,617 | 20,296 | 8,268 |
| 3 | 89,527 | 27,816 | 7,981 |
| 4 | 158,254 | 35,089 | 9,515 |
| 5 | 195,555 | 25,233 | 6,365 |
| 6 | 49,531 | 38,667 | 5,575 |
| 7 | 197,755 | 24,483 | 12,838 |
| 8 | 260,928 | 46,080 | 8,077 |
| 9 | 350,128 | 38,614 | 5,235 |
| 10 | 160,627 | 32,447 | 5,117 |
| 11 | 120,869 |  | 4,204 |
| 12 | 303,731 |  |  |
| 13 | 93,294 |  |  |
| 14 | 179,773 |  |  |
| 15 | 337,233 |  |  |
| 16 | 390,005 |  |  |
| 17 | 427,910 |  |  |
| 18 | 269,054 |  |  |
| 19 | 370,441 |  |  |
| 20 | 118,278 |  |  |
| 21 | 365,667 |  |  |
| 22 | 296,823 |  |  |
| **Totals** | **4,869,825** | **312,673** | **79,619** |
