# Supplementary figures and images for: Defining ecological regions in Italy based on a multivariate clustering approach: A first step towards a targeted vector borne disease surveillance
Source: PLoS One. 2019 Jul 3;14(7):e0219072. doi: 10.1371/journal.pone.0219072 (PMC6608978; doi:10.1371/journal.pone.0219072)

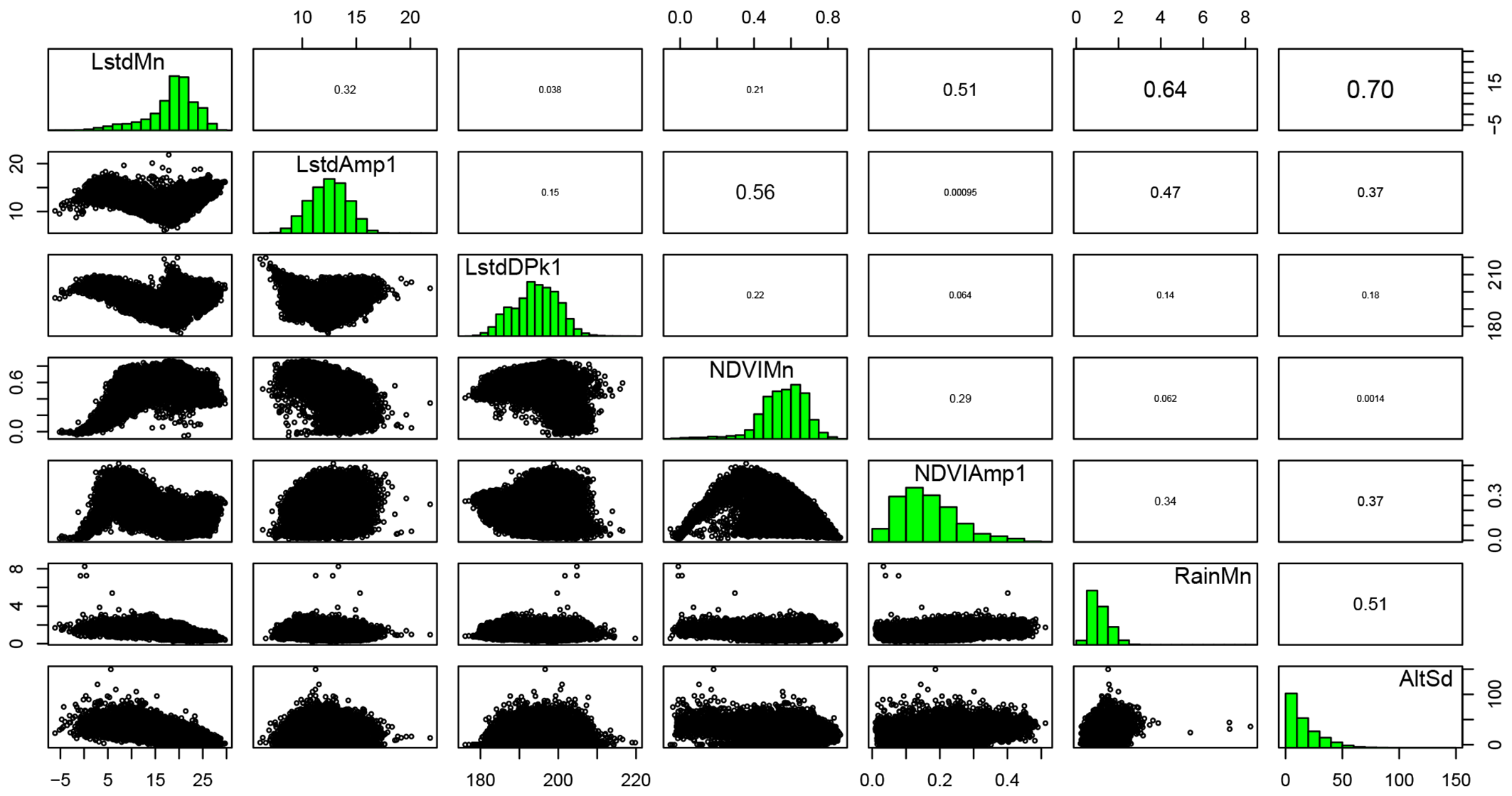

Supplement: S1 Fig — (TIF) [file pone.0219072.s006.tif]

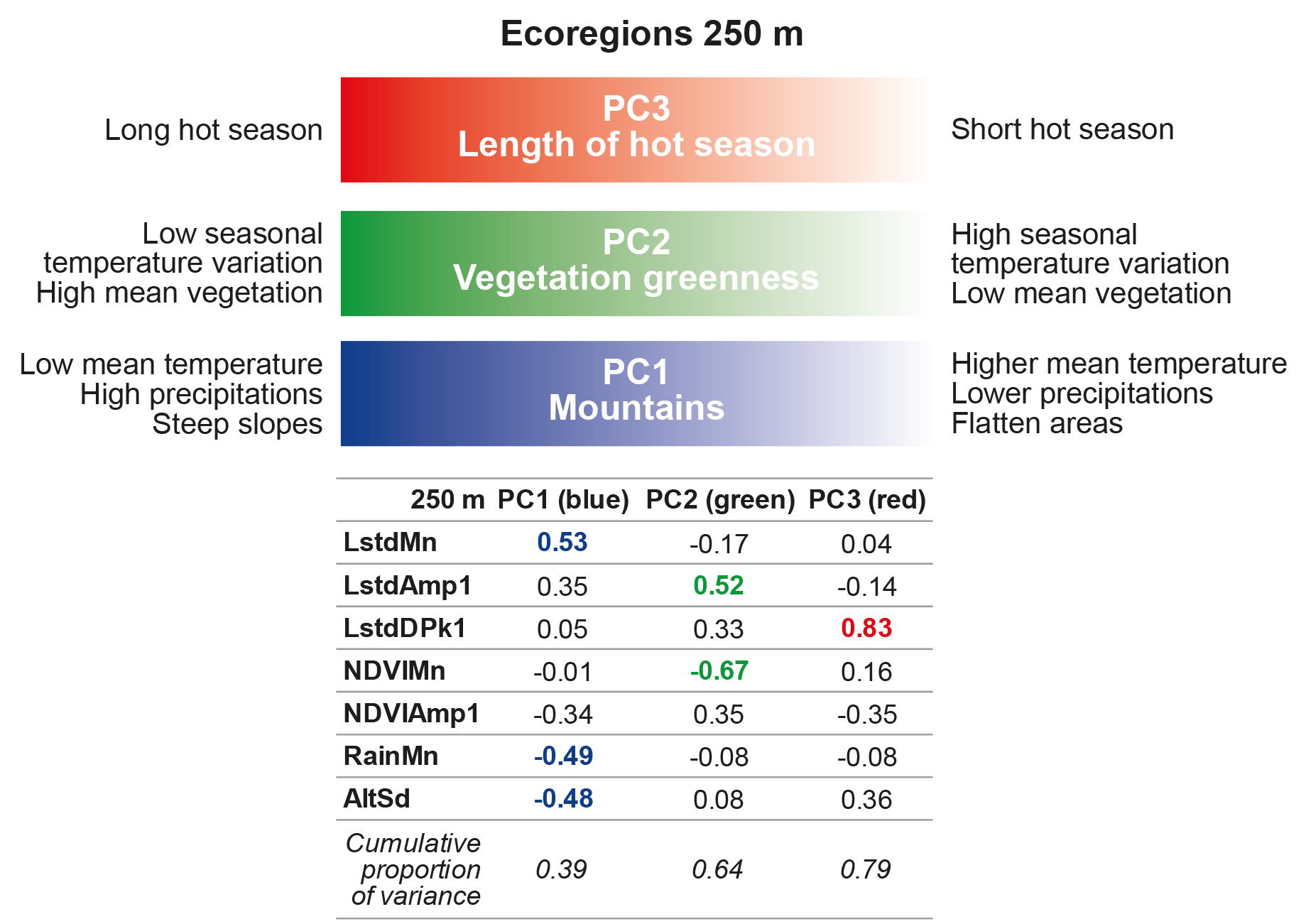

Supplement: S2 Fig — (TIF) [file pone.0219072.s007.tif]

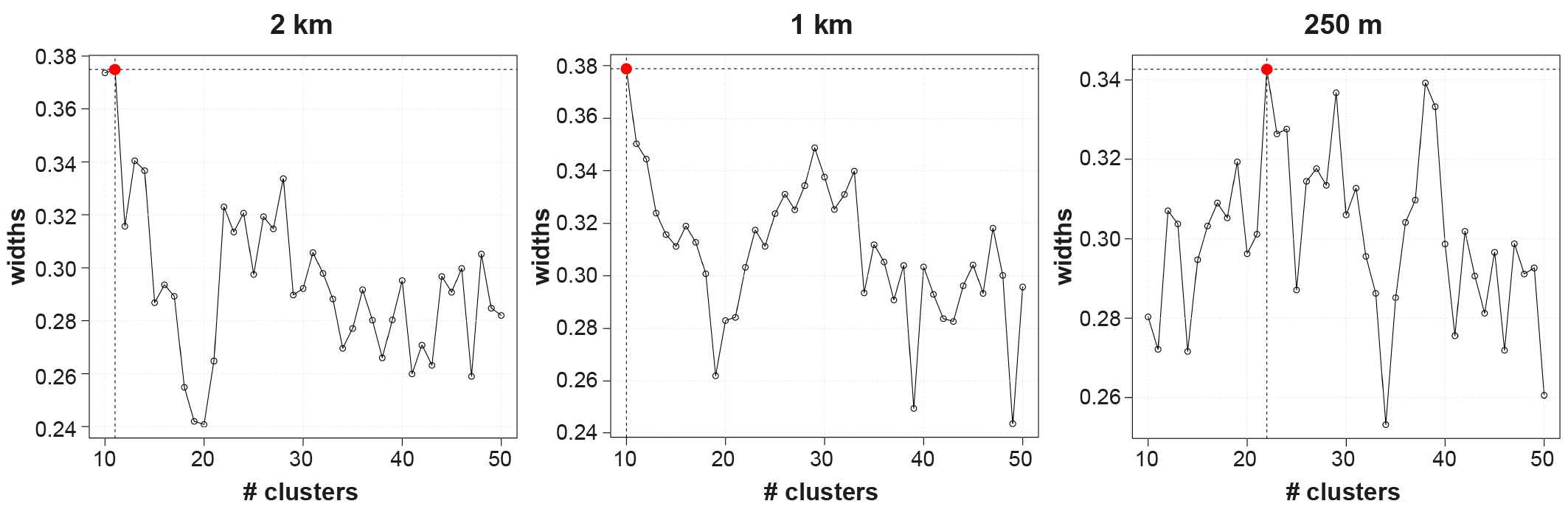

Supplement: S3 Fig — (TIF) [file pone.0219072.s008.tif]

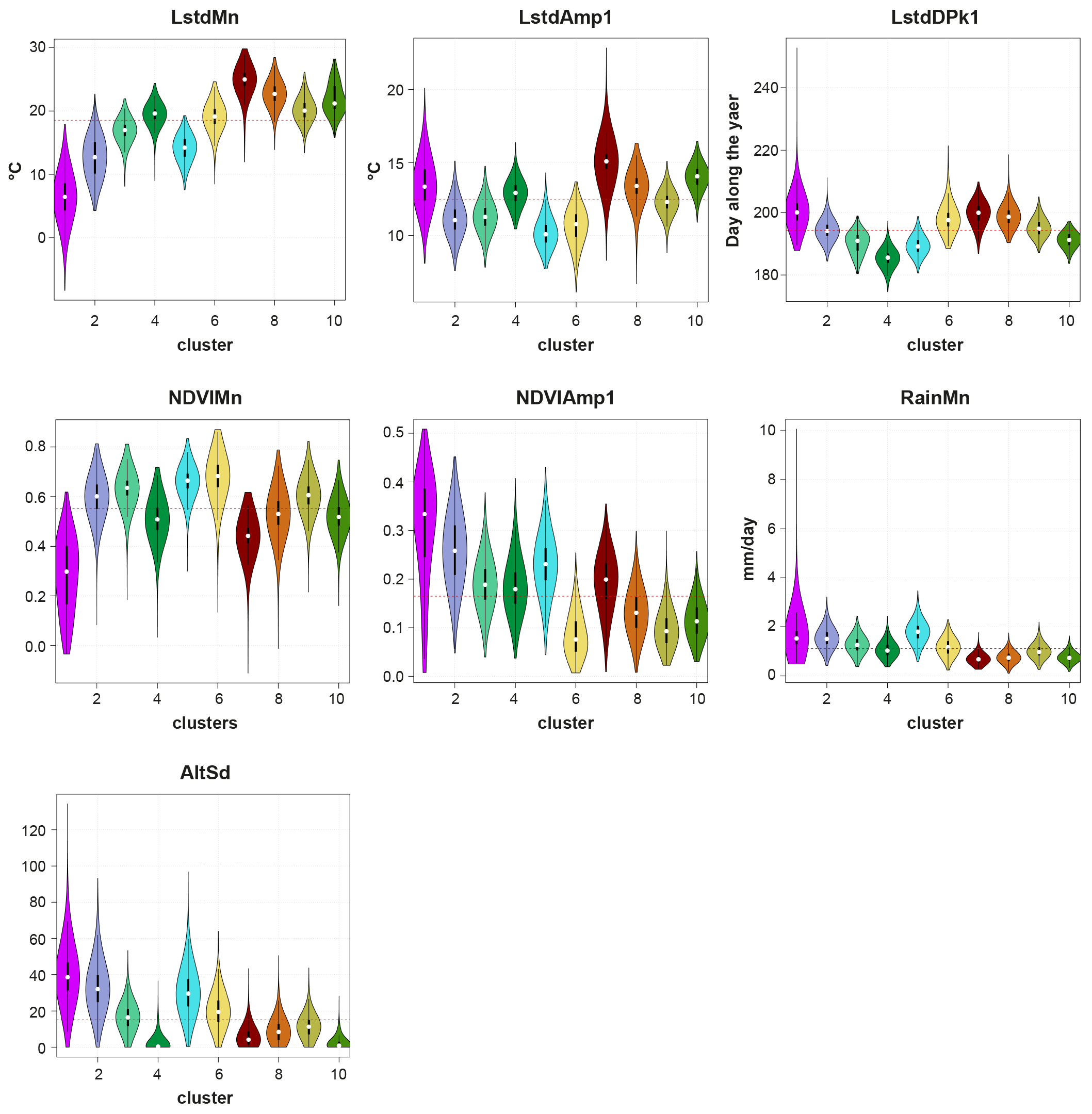

Supplement: S4 Fig — The violin plots show the probability density of the data at different values; they include a marker (white point) for the median of the data and a box indicating the interquartile range, with a kernel density overlaid. The red line reports the average value across all clusters. Each violin has the same color of the corresponding ecoregion. (TIF) [file pone.0219072.s009.tif]

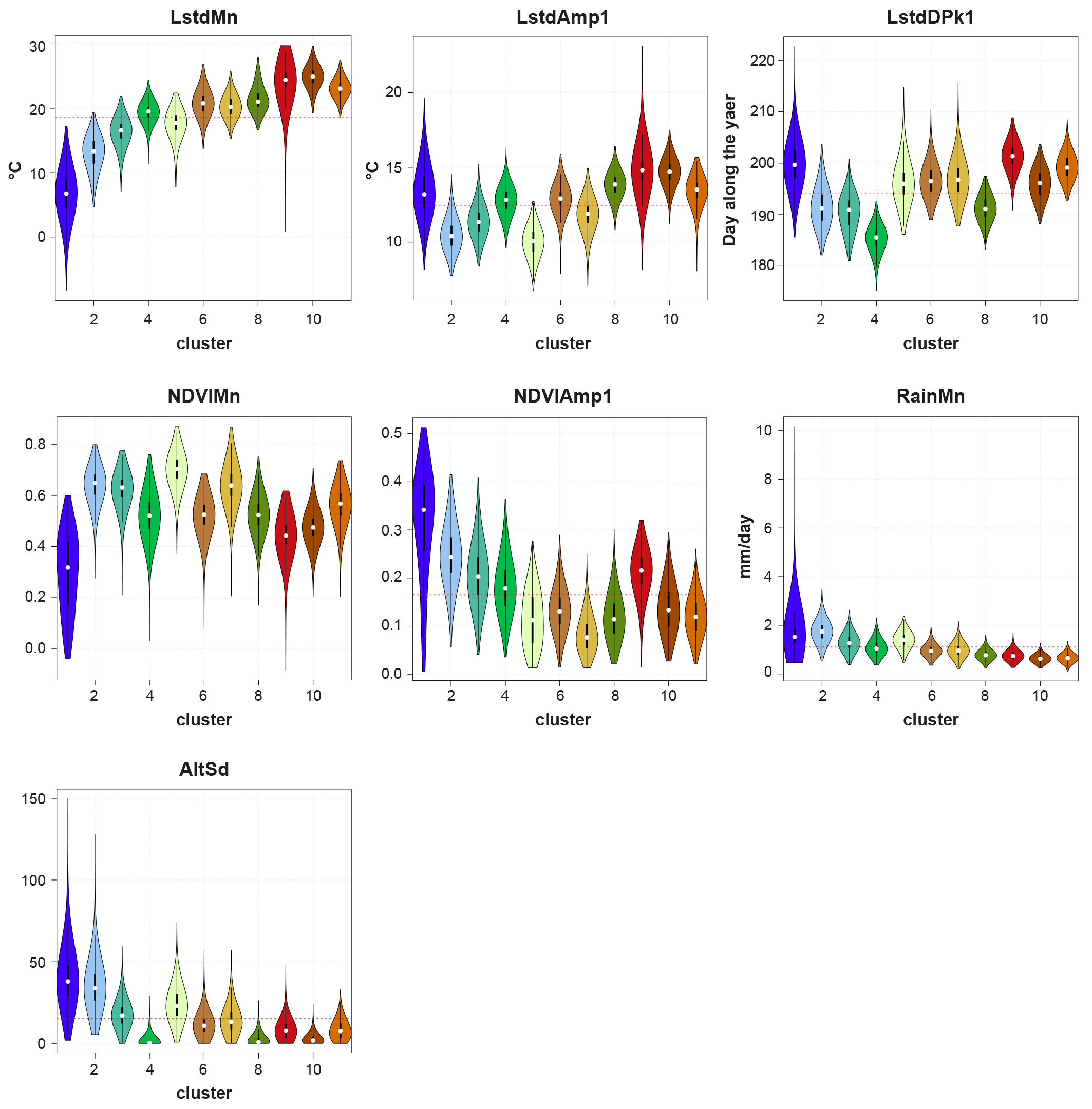

Supplement: S5 Fig — The violin plots show the probability density of the data at different values; they include a marker (white point) for the median of the data and a box indicating the interquartile range, with a kernel density overlaid. The red line reports the average value across all clusters. Each violin has the same color of the corresponding ecoregion. (TIF) [file pone.0219072.s010.tif]
